# Persistence of Pneumococcal Carriage among Older Adults in the Community despite COVID-19 Mitigation Measures

**DOI:** 10.1128/spectrum.04879-22

**Published:** 2023-04-10

**Authors:** Anne L. Wyllie, Sidiya Mbodj, Darani A. Thammavongsa, Maikel S. Hislop, Devyn Yolda-Carr, Pari Waghela, Maura Nakahata, Anne E. Stahlfeld, Noel J. Vega, Anna York, Orchid M. Allicock, Geisa Wilkins, Andrea Ouyang, Laura Siqueiros, Yvette Strong, Kelly Anastasio, Ronika Alexander-Parrish, Adriano Arguedas, Bradford D. Gessner, Daniel M. Weinberger

**Affiliations:** a Department of Epidemiology of Microbial Diseases, Yale School of Public Health, New Haven, Connecticut, USA; b Yale Center for Clinical Investigation, New Haven, Connecticut, USA; c Medical and Scientific Affairs, Pfizer Inc, Collegeville, Pennsylvania, USA; Keck School of Medicine of the University of Southern California

**Keywords:** pneumococcus, saliva, surveillance, carriage, COVID-19 pandemic

## Abstract

Reported rates of invasive pneumococcal disease were markedly lower than normal during the 2020/2021 winter in the Northern Hemisphere, the first year after the start of the COVID-19 pandemic. However, little is known about rates of carriage of pneumococcus among adults during this period. Between October 2020-August 2021, couples in the Greater New Haven Area, USA, were enrolled if both individuals were aged 60 years and above and did not have any individuals under the age of 60 years living in the household. Saliva samples and questionnaires regarding social activities and contacts and medical history were obtained every 2 weeks for a period of 10 weeks. Following culture-enrichment, extracted DNA was tested using qPCR for pneumococcus-specific sequences *piaB* and *lytA*. Individuals were considered positive for pneumococcal carriage when Ct values for *piaB* were ≤40. Results. We collected 567 saliva samples from 95 individuals (47 household pairs and 1 singleton). Of those, 7.1% of samples tested positive for pneumococcus, representing 22/95 (23.2%) individuals and 16/48 (33.3%) households. Study participants attended few social events during this period. However, many participants continued to have regular contact with children. Individuals who had regular contact with preschool and school-aged children (i.e., 2 to 9 year olds) had a higher prevalence of carriage (15.9% versus 5.4%). Despite COVID-19-related disruptions, a large proportion of older adults continued to carry pneumococcus. Prevalence was particularly high among those who had contact with school-aged children, but carriage was not limited to this group.

**IMPORTANCE** Carriage of Streptococcus pneumoniae (pneumococcus) in the upper respiratory tract is considered a prerequisite to invasive pneumococcal disease. During the first year of the COVID-19 pandemic, markedly lower rates of invasive pneumococcal disease were reported worldwide. Despite this, by testing saliva samples with PCR, we found that older adults continued to carry pneumococcus at pre-pandemic levels. Importantly, this study was conducted during a period when transmission mitigation measures related to the COVID-19 pandemic were in place. However, our observations are in line with reports from Israel and Belgium where carriage was also found to persist in children. In line with this, we observed that carriage prevalence was particularly high among the older adults in our study who maintained contact with school-aged children.

## INTRODUCTION

Mitigation measures that have been used to reduce the burden of COVID-19 have also had a profound effect on the incidence of disease caused by other pathogens. Major respiratory viruses, including influenza, respiratory syncytial virus, and human metapneumovirus, largely disappeared as causes of disease during the 2020-21 winter season in the Northern hemisphere (1–3). Invasive pneumococcal disease (IPD) declined sharply in the spring of 2020 across all age groups and did not return to near regular levels until spring or summer of 2021 (2, 4–6).

It was initially assumed that the reduction in the incidence of IPD was due to reduced transmission of the bacteria resulting from the implementation of non-pharmaceutical interventions. Pneumococcus is commonly carried in the upper respiratory tract of young children, however, the prevalence of pneumococcal carriage in children was near normal levels during 2020 to 2021 (3, 7). This demonstrates that children were still being exposed to and acquiring pneumococcus but not getting sick. Therefore, the decline in IPD observed in children might instead be related to the absence of infection by common respiratory viruses, which are thought to increase the risk of severe pneumococcal disease (3). Additionally, some of the decline in IPD in children and adults could also be related to reduced health care seeking or changes in diagnostic practices during the pandemic (8).

While transmission of pneumococcus among children continued at high levels during the first year of the pandemic, social distancing and other mitigation measures could have reduced the amount of contact and transmission from children to adults. Children are a major source of exposure of pneumococcus for the adult population (9–11), which was most evident from the sharp drop in pneumococcal disease in adults that occurred following the introduction of pneumococcal conjugate vaccines in children (12). Therefore, we expected that reductions in contact between children and adults might have occurred during the COVID-19 pandemic, leading to some of the reduction in IPD observed in adult populations. We evaluated carriage rates among adults ≥60 years of age living in the community in the United States during 2020 to 21 who were participating in an ongoing longitudinal carriage study. Detailed information about their activities and contacts, along with their carriage status, provides important insights into the potential drivers of pneumococcal epidemiology in older adults.

## RESULTS

### Population characteristics.

From October 30^th^, 2020 through June 2021, 95 individuals from 48 households were sampled and completed all 6 visits. One household was composed of a single individual who was enrolled into the study due to residing in a living facility for older adults with high levels of contact between residents; due to the pandemic-related community restrictions in place, we were unable to enroll more individuals from the same facility. The mean age was 71 years (range 60 to 86) (Fig. S1). Of the 570 samples collected, 3 were not tested due to low collection volume (*n* = 1) or a weather-related delay (2 weeks) in transporting samples to the lab (*n* = 2). Of the study participants, 72% were white, and 42% had a bachelor’s degree or higher. Eight individuals had a positive test for SARS-CoV-2 prior to enrollment in the study. None of the participants reported a positive test for SARS-CoV-2 while enrolled in the study nor tested positive for SARS-CoV-2 in any of the collected samples.

### Prevalence of pneumococcal carriage.

Overall, 40/567 (7.1%) of samples tested positive for pneumococcus based on *piaB*, with 22/95 (23.2%) individuals colonized on at least 1 time point (Fig. S2). Several individuals were colonized at multiple time points, including 2 individuals (participants 3 and 41) who were colonized throughout the 10 weeks sampling period and a third who was colonized at 5 of the 6 time points (participant 33). In 6/48 (12.5%) households, both members were carriers, though not necessarily at the same time point.

When samples were positive for both *piaB* and *lytA* (*n* = 34), there was good concordance in the bacterial density (Ct value) (Fig. S3). In some samples, the concentration of *lytA* was higher than *piaB* (lower Ct), likely reflecting the presence of non-pneumococcal *Streptococci* spp. in addition to pneumococcus (Fig. S3) (13, 14). In addition to higher specificity, the sensitivity of the *piaB* assay was also slightly higher, resulting in some samples near the limit of detection that were positive for *piaB* and negative for *lytA* (Fig. S3). It is for these reasons that when determining sample positivity, we relied on *piaB* alone.

To confirm the qPCR results, we used traditional culture-based methods, which are feasible when a high concentration of pneumococcus is detected (15). From the 40 samples that tested positive for *piaB*, 5 isolates, all from the same individual (participant 41), were successfully isolated and identified as pneumococcus. Each of the isolates was optochin sensitive, and the presence of both *piaB* and *lytA* genes were confirmed by qPCR. All isolates were identified as serotype 15B/C by latex agglutination.

### Reported activities outside of the home.

During this period, which included the first winter of the COVID-19 pandemic, participants continued to take part in some social activities outside the home (Table 1). The most commonly reported activities were gathering with family (40%) and friends (29%). Just 6% of participants reported taking part in activities at a community center, and 8% participated in fitness activities. The prevalence of pneumococcal carriage was modestly higher among those who reported activities with family (13.5%) compared with those participating in other social activities or those reporting no social activity (8.1%).

**TABLE 1:** Relationship between pneumococcal detection and activities outside the home during the previous 2 weeks

Activities outside the home	*N* ^*a*^	Pneumococcus (*piaB*+)	Percent positive
Activities with family	74	10	13.5%
Activities with friends	38	0	0%
Fitness activities	13	0	0%
Activities at community centers	8	0	0%
Other social activity	115	5	4.3%
Total*	579	40	6.9%

a Numbers of visits at which people reported these activities. Participants were only asked about recent activities at visits 2 to 6.

### Prevalence is higher among those in contact with children.

**FIG 1:**
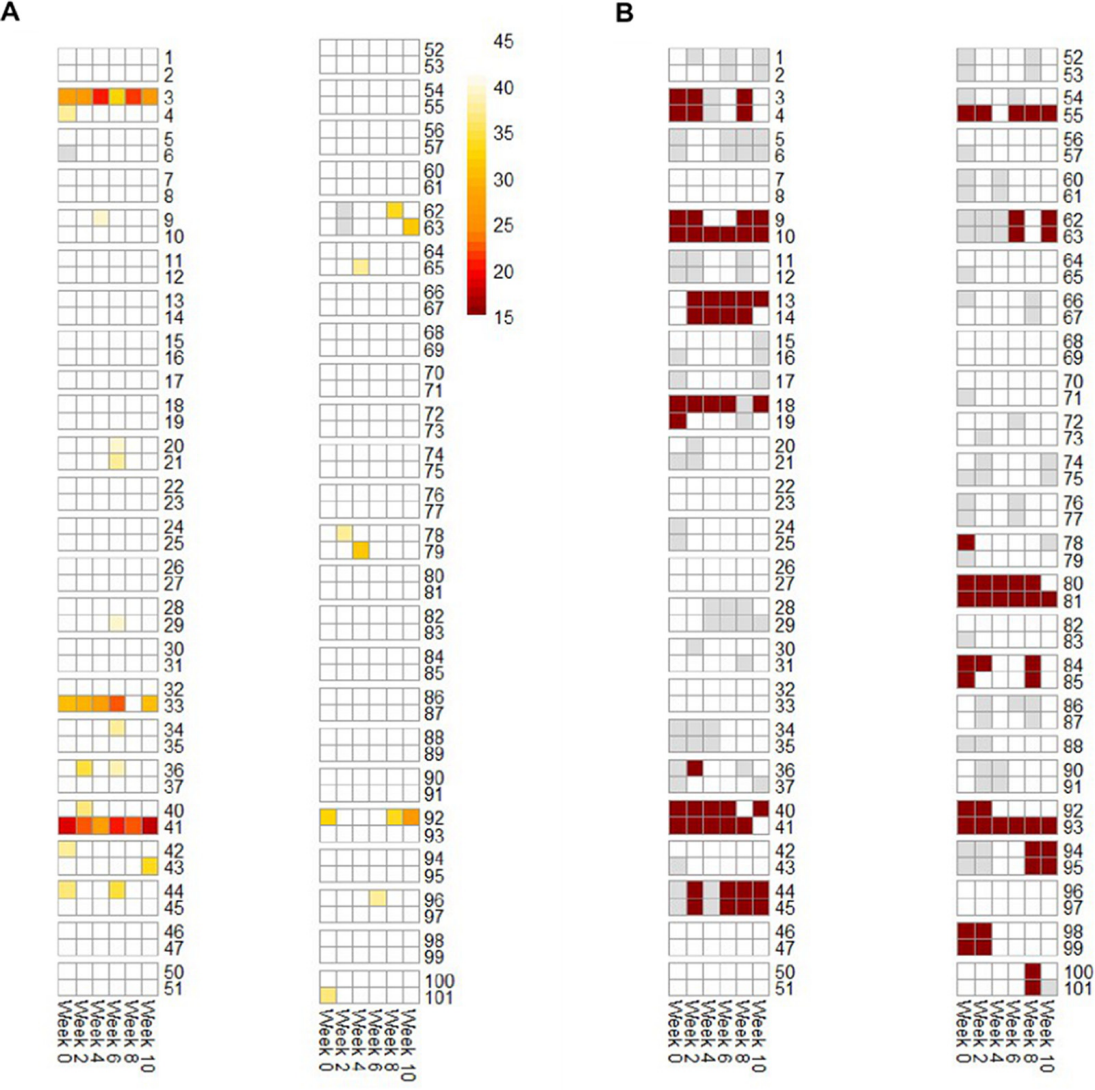
(A) Detection of pneumococcus as measured by Ct values from PCR assays targeting pneumococcal gene, *piaB*. Colored boxes indicate the individual is colonized, with darker colors indicating a higher presence of pneumococcus (lower Ct values). (B) Contact with children. Maroon indicates the individual reported contact with children, white indicates no reported contacts, gray indicates the question was not answered or the survey is missing.

The prevalence of pneumococcal carriage was substantially higher among individuals who had contact with children (13.0% versus 3.5%, *P = *0.002) (Fig. 1 and Table 2). Participants who reported recent contact with <5 year olds and 5 to 9 year olds had elevated prevalence (17.5%, *P = *0.001; 15.9%, *P = *0.007, respectively) (Table 2). Prevalence was not notably higher among those reporting contact with children older than 10 years of age (10.4%, *P = *0.47). While the numbers are sparse, further subdividing the <5 year old population demonstrates progressively higher prevalence among those reporting contact with children <12m (11.9%), 12 to 23 months (13.3%) and 24 to 59 months (20.5%).

**TABLE 2:** Relationship between pneumococcal detection and contact with children

Contact with children	*N*	Pneumococcus (*piaB*+)	Percent positive
Contact with any children			
No contact	318	11	3.5%
Contact	192	25	13.0%
Missing	69	4	5.8%
Contact <5 yr old children			
No contact	443	23	5.2%
Contact	80	14	17.5%
Missing	56	3	5.4%
Contact 5 to 10 yr old children			
No contact	441	24	5.4%
Contact	82	13	15.9%
Missing	56	3	5.4%
Contact >10 yr old children			
No contact	456	30	6.6%
Contact	67	7	10.4%
Missing	56	3	5.4%

## DISCUSSION

Despite sharp declines in reported rates of IPD among adults during the first winter season of the COVID-19 pandemic (2020 to 21), older adults residing in the community continued to carry pneumococcus at levels consistent with what has been seen in other pre-pandemic studies of older adults that used similar molecular methods (16, 17). However, this study was conducted during a period when a reversion to stricter transmission mitigation measures was implemented throughout the state of Connecticut due to a resurgence in COVID-19 cases (18, 19). In the Greater New Haven Area, mask mandates were enforced in public spaces and had high adherence (20), and usual community activities were canceled throughout the study period. Study participants reported few activities outside of the home, even as restrictions were eased in March 2021 (21). However, many individuals did continue to have regular contact with their families, including young children, and these participants had particularly high rates of carriage. This is consistent with studies conducted among younger adults that children are a major driver of transmission of pneumococcus in the community (22, 23).

In this study setting, the period prevalence of pneumococcal carriage detected in this population of ≥60 year olds in the Greater New Haven Area was 23.2%. While other carriage studies conducted in older adults, also in the US, have reported lower rates of carriage (24), the difference in carriage rates can likely be attributed to sample and testing methodologies. As observed in the current study, saliva-based approaches and those that use qPCR following culture-enrichment tend to have higher sensitivity than those based on swabs and culture alone (15, 16). By including oropharyngeal swabs in addition to nasopharyngeal swabbing, Branche et al. reported a similar longitudinal carriage rate (≥25%), though did not detect an effect of contact with children (17). In their analyses however, Branche et al. considered contact with any children under 5 years of age (17) and as our analyses show, children under 2 years of age contribute little to transmission (25). This is also reflected in reports on their little contribution to disease incidence in older adults (25). When contact with children is not stratified by age group, and the majority of child contacts are younger, the effect of transmission is harder to detect. In this study, we asked study participants the ages of children with whom they interacted to enable these stratified analyses.

When surveying pneumococcal carriage using saliva samples, 1 limitation is the difficulty in isolating individual pneumococci due to the highly polymicrobial nature of saliva. Plating saliva on agar plates results in a solid lawn of diverse, bacterial growth. Even when heavily diluting the saliva prior to plating, pneumococcus typically comprises a minority. This greater abundance of growth compared to both nasopharyngeal and oropharyngeal swabs can make it near impossible to isolate individual colonies (22, 26). Thus, for the detection of pneumococcus we must rely on alternative methods. The method employed in the current study, testing DNA extracted from culture-enriched saliva in qPCR for pneumococcus genes *piaB* and *lytA* is one that has been used in previous studies (13, 16, 22, 27) and an approach that has demonstrated high sensitivity compared to other methods (28). However, we classified samples as positive for pneumococcus based on the detection of *piaB* alone, without a requirement for positivity of also *lytA* (29). While *lytA* has quickly become considered the gold standard gene target for the molecular detection of pneumococcus, there have been a growing number of reports of false positivity in this assay (30–32), predominantly due to the higher presence of closely related, non-pneumococcus Streptococcus spp. in oral samples which carry pneumococcal gene homologues (13, 14, 33). This was reflected in many samples that generated a large discrepancy in the Ct values reported for these two gene targets (Fig. S3). Additionally, despite numerous modifications, in our study setting, the sensitivity of the *lytA* qPCR assay was less than that of the *piaB* assay. This meant that there were instances toward the limit of detection of the qPCR assay where samples were positive for only *piaB*, which we confirmed with re-testing when the *piaB* Ct value was >35. Samples were only called positive if they tested positive twice or 2 out of 3 times in a tie-breaker situation. The limitation of relying upon only *piaB* is that *piaB* is missing from nontypeable pneumococci (34, 35) as well as in a few reported encapsulated strains (31). While our approach may have classified such samples as negative for pneumococcus, we expect these to rarely occur in this population of older adults, and if present, would mean we have a reported a conservative estimate of the true carriage rates.

In this first study season of an ongoing study investigating rates of pneumococcal carriage in Greater New Haven, we found little evidence of impact of pandemic mitigation measures on rates of the carriage of pneumococcus in older adults. During this period of reduced social contact, study results suggest school-aged children are the likely source of continued presence of pneumococcus in most study subjects. Importantly, the high rate of nonspecific signal detected in the widely used *lytA* qPCR assay demonstrates the importance of targeting multiple gene targets for reliable and specific detection of pneumococcus in oral samples (13). Follow-up studies with molecular serotyping of collected samples will provide greater insight into this observation and into transmission patterns of pneumococcus within households consisting of only individuals over the age of 60 years.

## MATERIALS AND METHODS

### Ethics.

This study was approved by the Institutional Review Board at Yale School of Medicine (protocol ID. #2000026100). Demographic data and samples were only collected after the study participant had acknowledged that they had understood the study protocol and provided verbal- or written-informed consent. All participant information and samples were collected in association with anonymized study identifiers.

### Enrolment and eligibility.

These data are drawn from the first sampling year of an ongoing study of pneumococcal carriage. The broader study is designed to quantify and detect rates of acquisition of pneumococcal carriage among older adults and the role of household transmission between cohabitating older adults. We recruited pairs of individuals residing in the community in the greater New Haven area who were both 60 years of age or older and who did not have anyone under the age of 60 years living in the household. If an individual had symptoms of respiratory illness at time of consenting or had received antibiotics or pneumococcal vaccination within the past 4 weeks, the enrollment of that household pair into the study was delayed by up to 4 weeks. There were no exclusion criteria based on underlying health status.

### Sample and data collection.

Household pairs were sampled every 2 weeks, for a total of 6 visits covering 10 weeks. At each visit, the participants provided a self-collected saliva sample (36) into an empty 25 mL Eppendorf conical tube and answered questions about their social activities, doctors’ visits, recent contact with children, and any respiratory symptoms they were experiencing or had experienced in the 2 weeks prior (Appendix). Study participants left their samples outside their front door for contactless-collection and transport back to the laboratory at room temperature.

### Sample processing and pneumococcal detection.

On arrival at the lab, 100 μL of raw saliva was plated on TSAII plates with 5% sheep’s blood and 10% gentamicin and grown overnight at 37°C with 5% CO_2_. Growth was harvested into 2100 μL of BHI supplemented with 10% glycerol and stored at –80°C until further processing. These samples were considered as culture-enriched for pneumococcus (16). DNA was extracted from 200 μL of each culture-enriched sample using the MagMAX Viral/Pathogen Nucleic Acid isolation kit (ThermoFisher Scientific) on the KingFisher Apex (ThermoFisher Scientific) with a modified protocol. Briefly, samples first underwent an extended digestion step, with 10 μL of proteinase K added to each sample and were incubated at 56°C for 10 min followed by heat inactivation of the proteinase K at 95°C for 10 min. Binding buffer and magnetic beads (25 μL) were added separately before proceeding with the KingFisher Apex extraction protocol which included an additional elution step, eluting extracted DNA into 2 plates of 50 μL of elution buffer. Purified DNA was tested by qPCR using primers and probes specific for 2 pneumococcal genes: *piaB* (15, 37) and *lytA* (38). The assays were carried out in 20 μL reaction volumes using SsoAdvanced Universal Probe Supermix (Bio-Rad, USA), 2.5 μL of genomic DNA and primer/probe mixes at concentrations of 250 nM (Iowa Black quenchers) for *piaB* (1 μL per reaction) and *lytA* (1.2 μL per reaction). DNA of S. pneumoniae serotype 19F was included as a positive control in every run. Assays were run on a CFX96 Touch (Bio-Rad) under the following conditions: 95°C for 3 min, followed by 45 cycles of 98°C for 15 s and 60°C for 30 s. Because many other *Streptococci* have the *lytA* gene, a sample was only considered to be positive for pneumococcus if it was positive for *piaB* (29). Samples were classified as positive with a *piaB* cycle threshold (Ct) value of <40 by RT-qPCR. However, DNA templates which generated a *piaB* Ct value between 35 and 40 were re-tested in qPCR. Only samples that tested positive twice (or twice out of 3 tests when a discrepant result occurred) were reported as a positive result.

### Strain isolation and serotyping.

Cultured-enriched saliva samples in which a higher concentration of pneumococcus was detected (<30 Ct by qPCR) were re-visited in an attempt to isolate pure pneumococcus. Samples were serially diluted 10-fold over a range of 10^−1^ to 10^−5^ in 1X PBS. The 10^−5^ and 10^−6^ serially diluted samples were plated (100 μL) onto plain blood agar plates. After overnight incubation, culture plates were visually screened for pneumococcal-like colonies. Each colony of pneumococcus-like morphology was streaked onto a plain blood agar plate and then inoculated into 50 μL of elution buffer (ThermoFisher Scientific) in a microcentrifuge tube. From each saliva sample, a total of 20 colonies were streaked onto one plain blood agar plate, with colonies pooled by 5 in each microcentrifuge tube. Pooled bacterial colonies were incubated for 10 min at 95°C on a heating block, then tested in qPCR for *piaB* and *lytA* to confirm the identity of the isolates. Streaked isolates from the pooled boilate samples that generated any signal <40 Ct for either *piaB* or *lytA* were individually tested for optochin susceptibility. Optochin susceptible colonies which tested positive for both *piaB* and *lytA* were then serotyped by latex agglutination (Statens Serum Institut) (39).

### Detection of SARS-CoV-2.

All saliva samples were also tested for the presence of SARS-CoV-2 virus RNA using the extraction-free SalivaDirect assay (40). Briefly, 50 μL of each sample was heated at 95°C for 5 min before being tested in RT-qPCR for SARS-CoV-2 (41).

### Statistical analysis.

Differences in the frequency of categorical outcomes were compared using Fisher’s Exact test in the R Statistical Software v4.1.2.

## Supplementary Material

Reviewer comments

## References

[1] Yuan H, Yeung A, Yang W. 2022. Interactions among common non-SARS-CoV-2 respiratory viruses and influence of the COVID-19 pandemic on their circulation in New York City. Influenza Other Respi Viruses 16:605–799. doi:10.1111/irv.12976. PMC9111828 35278037

[2] Brueggemann AB, van Rensburg MJ, Shaw D, McCarthy ND, Jolley KA, Maiden MCJ, van der Linden MPG, Amin-Chowdhury Z, Bennett DE, Borrow R, Brandileone MC, Broughton K, Campbell R, Cao B, Casanova C, Choi EH, Chu YW, Clark SA, Claus H, Coelho J, Corcoran M, Cottrell S, Cunney RJ, Dalby T, Davies H, de Gouveia L, Deghmane AE, Demczuk W, Desmet S, Drew RJ, Du Plessis M, Erlendsdottir H, Fry NK, Fuursted K, Gray SJ, Henriques-Normark B, Hale T, Hilty M, Hoffmann S, Humphreys H, Ip M, Jacobsson S, Johnston J, Kozakova J, Kristinsson KG, Krizova P, Kuch A, Ladhani SN, Lâm TT, Lebedova V, et al. 2021. Changes in the incidence of invasive disease due to *Streptococcus pneumoniae*, *Haemophilus influenzae*, and *Neisseria meningitidis* during the COVID-19 pandemic in 26 countries and territories in the Invasive Respiratory Infection Surveillance Initiative: a prospective analysis of surveillance data. Lancet Digital Health 3:E360–E370. doi:10.1016/S2589-7500(21)00077-7. 34045002 PMC8166576

[3] Danino D, Ben-Shimol S, Van Der Beek BA, Givon-Lavi N, Avni YS, Greenberg D, Weinberger DM, Dagan R. 2021. Decline in pneumococcal disease in young children during the COVID-19 pandemic in Israel associated with suppression of seasonal respiratory viruses, despite persistent pneumococcal carriage: a prospective cohort study. Clin Infect Dis 75:e1154–e1164. doi:10.1093/cid/ciab1014. PMC8754767 34904635

[4] Casanova C, Küffer M, Leib SL, Hilty M. 2021. Re-emergence of invasive pneumococcal disease (IPD) and increase of serotype 23B after easing of COVID-19 measures, Switzerland, 2021. Emerg Microbes Infect 10:2202–2204. doi:10.1080/22221751.2021.2000892. 34723783 PMC8648035

[5] Perniciaro S, van der Linden M, Weinberger DM. 2022. Re-emergence of invasive pneumococcal disease in Germany during the Spring and Summer of 2021. Clin Infect Dis 75:1149–1153. doi:10.1093/cid/ciac100. 35136983 PMC9383454

[6] McNeil JC, Flores AR, Kaplan SL, Hulten KG. 2021. The indirect impact of the SARS-CoV-2 pandemic on invasive group a *Streptococcus*, *Streptococcus pneumoniae* and *Staphylococcus aureus* infections in Houston area children. Pediatr Infect Dis J 40:e313–e316. doi:10.1097/INF.0000000000003195. 34250979 PMC8279221

[7] Willen L, Ekinci E, Cuypers L, Theeten H, Desmet S. 2021. Infant pneumococcal carriage in Belgium not affected by COVID-19 containment measures. Front Cell Infect Microbiol 11:825427. doi:10.3389/fcimb.2021.825427. 35111700 PMC8801737

[8] Dirkx KKT, Mulder B, Post AS, Rutten MH, Swanink CMA, Wertheim HFL, Cremers AJH. 2021. The drop in reported invasive pneumococcal disease among adults during the first COVID-19 wave in the Netherlands explained. Int J Infect Dis 111:196–203. doi:10.1016/j.ijid.2021.08.060. 34455081 PMC8444629

[9] Flasche S, Lipsitch M, Ojal J, Pinsent A. 2020. Estimating the contribution of different age strata to vaccine serotype pneumococcal transmission in the pre vaccine era: a modelling study. BMC Med 18:129. doi:10.1186/s12916-020-01601-1. 32517683 PMC7285529

[10] Weinberger DM, Pitzer VE, Regev-Yochay G, Givon-Lavi N, Dagan R. 2019. Association between the decline in pneumococcal disease in unimmunized adults and vaccine-derived protection against colonization in toddlers and preschool-aged children. Am J Epidemiol 188:160–168. doi:10.1093/aje/kwy219. 30462150 PMC6321804

[11] Walter ND, Taylor TH, Dowell SF, Mathis S, Moore MR, Active Bacterial Core Surveillance System Team. 2009. Holiday spikes in pneumococcal disease among older adults. N Engl J Med 361:2584–2585. doi:10.1056/NEJMc0904844. 20032333

[12] Pilishvili T, Lexau C, Farley MM, Hadler J, Harrison LH, Bennett NM, Reingold A, Thomas A, Schaffner W, Craig AS, Smith PJ, Beall BW, Whitney CG, Moore MR, Active Bacterial Core Surveillance/Emerging Infections Program Network. 2010. Sustained reductions in invasive pneumococcal disease in the era of conjugate vaccine. J Infect Dis 20:32–41. doi:10.1086/648593. 19947881

[13] Hislop MS, Allicock OM, Thammavongsa DA, Mbodj S, Nelson A, Shaw AC, Weinberger DM, Wyllie AL. 2022. High levels of detection of non-pneumococcal species of *Streptococcus* in saliva from adults in the USA. bioRxiv. https://www.medrxiv.org/content/10.1101/2022.11.20.22282557v1. 10.1128/spectrum.05207-22 PMC10269540 37067447

[14] Whatmore AM, Efstratiou A, Pickerill AP, Broughton K, Woodard G, Sturgeon D, George R, Dowson CG. 2000. Genetic relationships between clinical isolates of *Streptococcus pneumoniae*, *Streptococcus oralis*, and *Streptococcus mitis*: characterization of “Atypical” pneumococci and organisms allied to *S. mitis* harboring *S. pneumoniae* virulence factor-encoding genes. Infect Immun 68:1374–1382. doi:10.1128/IAI.68.3.1374-1382.2000. 10678950 PMC97291

[15] Trzciński K, Bogaert D, Wyllie A, Chu MLJN, van der Ende A, Bruin JP, van den Dobbelsteen G, Veenhoven RH, Sanders EAM. 2013. Superiority of trans-oral over trans-nasal sampling in detecting *Streptococcus pneumoniae* colonization in adults. PLoS One 8:e60520. doi:10.1371/journal.pone.0060520. 23555985 PMC3610877

[16] Krone CL, Wyllie AL, van Beek J, Rots NY, Oja AE, Chu MLJN, Bruin JP, Bogaert D, Sanders EAM, Trzciński K. 2015. Carriage of *Streptococcus pneumoniae* in aged adults with influenza-like-illness. PLoS One 10:e0119875. doi:10.1371/journal.pone.0119875. 25789854 PMC4366201

[17] Branche AR, Yang H, Java J, Holden-Wiltse J, Topham DJ, Peasley M, Frost MR, Nahm MH, Falsey AR. 2018. Effect of prior vaccination on carriage rates of *Streptococcus pneumoniae* in older adults: a longitudinal surveillance study. Vaccine 36:4304–4310. doi:10.1016/j.vaccine.2018.05.107. 29871816

[18] OxCGRT. OxCGRT_US_subnational_05Aug2020.Csv at master · OxCGRT/USA-covid-policy. Github. https://github.com/OxCGRT/USA-covid-policy. Accessed 4 February 2023.

[19] PHASE 1 REOPENING. Connecticut – back to business – COVID-19 phase I, phase II and phase III reopening rules and executive orders. https://www.hklaw.com/-/media/files/stateorders/ctbacktobusinesssectorrulesphase123.pdf?la=en. Accessed 4 February 2023.

[20] Delphi Group. Delphi’s COVID-19 trends and impact surveys (CTIS). https://delphi.cmu.edu/covid19/ctis/. Accessed 18 March 2022.

[21] City of New Haven Health Department. NHVHealth 2021 annual report. 2021. https://www.newhavenct.gov/home/showpublisheddocument/16233/638001376291470000. Accessed 4 February 2023.

[22] Wyllie AL, Rümke LW, Arp K, Bosch AATM, Bruin JP, Rots NY, Wijmenga-Monsuur AJ, Sanders EAM, Trzciński K. 2016. Molecular surveillance on *Streptococcus pneumoniae* carriage in non-elderly adults; little evidence for pneumococcal circulation independent from the reservoir in children. Sci Rep 6:34888. doi:10.1038/srep34888. 27713565 PMC5054371

[23] Melegaro A, Choi Y, Pebody R, Gay N. 2007. Pneumococcal carriage in United Kingdom families: estimating serotype-specific transmission parameters from longitudinal data. Am J Epidemiol 166:228–235. doi:10.1093/aje/kwm076. 17517684

[24] Milucky J, Carvalho M de G, Rouphael N, Bennett NM, Talbot HK, Harrison LH, Farley MM, Walston J, Pimenta F, Lessa FC, Adult Pneumococcal Carriage Study Group. 2019. *Streptococcus pneumoniae* colonization after introduction of 13-valent pneumococcal conjugate vaccine for US adults 65 years of age and older, 2015–2016. Vaccine 37:1094–1100. doi:10.1016/j.vaccine.2018.12.075. 30685247 PMC6371770

[25] Wyllie AL, Warren JL, Regev-Yochay G, Givon-Lavi N, Dagan R, Weinberger DM. 2019. Serotype patterns of pneumococcal disease in adults are correlated with carriage patterns in older children. medRxiv. doi:10.1101/2019.12.18.19015180.. PMC8315131 32989457

[26] Wyllie AL, Chu MLJN, Schellens MHB, van Engelsdorp Gastelaars J, Jansen MD, van der Ende A, Bogaert D, Sanders EAM, Trzciński K. 2014. *Streptococcus pneumoniae* in saliva of Dutch primary school children. PLoS One 9:e102045. doi:10.1371/journal.pone.0102045. 25013895 PMC4094488

[27] Almeida ST, Paulo AC, Froes F, de Lencastre H, Sá-Leão R. 2021. Dynamics of pneumococcal carriage in adults: a new look at an old paradigm. J Infect Dis 223:1590–1600. doi:10.1093/infdis/jiaa558. 32877517

[28] Satzke C, Dunne EM, Porter BD, Klugman KP, Kim Mulholland E, PneuCarriage project group. 2015. The PneuCarriage Project: a multi-centre comparative study to identify the best serotyping methods for examining pneumococcal carriage in vaccine evaluation studies. PLoS Medicine 12:e1001903. doi:10.1371/journal.pmed.1001903. 26575033 PMC4648509

[29] Miellet WR, van Veldhuizen J, Nicolaie MA, Mariman R, Bootsma HJ, Bosch T, Rots NY, Sanders EAM, van Beek J, Trzciński K. 2021. Influenza-like illness exacerbates pneumococcal carriage in older adults. Clin Infect Dis 73:e2680–e2689. doi:10.1093/cid/ciaa1551. 33124669

[30] Simões AS, Tavares DA, Rolo D, Ardanuy C, Goossens H, Henriques-Normark B, Linares J, de Lencastre H, Sá-Leão R. 2016. lytA-based identification methods can misidentify *Streptococcus pneumoniae*. Diagn Microbiol Infect Dis 85:141–148. doi:10.1016/j.diagmicrobio.2016.03.018. 27107535

[31] Tavares DA, Handem S, Carvalho RJ, Paulo AC, de Lencastre H, Hinds J, Sá-Leão R. 2019. Identification of *Streptococcus pneumoniae* by a real-time PCR assay targeting SP2020. Sci Rep 9:3285. doi:10.1038/s41598-019-39791-1. 30824850 PMC6397248

[32] Boelsen LK, Dunne EM, Gould KA, Ratu FT, Vidal JE, Russell FM, Mulholland EK, Hinds J, Satzke C. 2020. The challenges of using oropharyngeal samples to measure pneumococcal carriage in adults. mSphere 5:e00478-20. doi:10.1128/mSphere.00478-20. 32727860 PMC7392543

[33] Greve T, Møller JK. 2012. Accuracy of using the lytA gene to distinguish *Streptococcus pneumoniae* from related species. J Med Microbiol 61:478–482. doi:10.1099/jmm.0.036574-0. 22135022

[34] Tavares DA, Simões AS, Bootsma HJ, Hermans PW, de Lencastre H, Sá-Leão R. 2014. Non-typeable pneumococci circulating in Portugal are of cps type NCC2 and have genomic features typical of encapsulated isolates. BMC Genomics 15:863. doi:10.1186/1471-2164-15-863. 25283442 PMC4200197

[35] Wyllie AL, Pannekoek Y, Bovenkerk S, van Engelsdorp Gastelaars J, Ferwerda B, van de Beek D, Sanders EAM, Trzciński K, van der Ende A. 2017. Sequencing of the variable region of rpsB to discriminate between *Streptococcus pneumoniae* and other streptococcal species. Open Biol 7:170074. doi:10.1098/rsob.170074. 28931649 PMC5627049

[36] Allicock OM, Petrone M, Yolda-Carr D, Breban M, Walsh H, Watkins AE, Rothman JE, Farhadian S, Grubaugh ND, Wyllie AL. Evaluation of saliva self-collection devices for SARS-CoV-2 diagnostics. 2021. https://papers.ssrn.com/abstract=3877563. Accessed 4 August 2021. 10.1186/s12879-022-07285-7 PMC8953967 35337266

[37] Wyllie AL, Wijmenga-Monsuur AJ, van Houten MA, Bosch AATM, Groot JA, van Engelsdorp Gastelaars J, Bruin JP, Bogaert D, Rots NY, Sanders EAM, Trzciński K. 2016. Molecular surveillance of nasopharyngeal carriage of *Streptococcus pneumoniae* in children vaccinated with conjugated polysaccharide pneumococcal vaccines. Sci Rep 6:23809. doi:10.1038/srep23809. 27046258 PMC4820691

[38] Mosadegh M, Asadian R, Emamie AD, Rajabpour M, Najafinasab E, Azarsa M. 2020. Impact of laboratory methods and gene targets on detection of *Streptococcus pneumoniae* in isolates and clinical specimens. Rep Biochem Mol Biol 9:216–222. doi:10.29252/rbmb.9.2.216. 33178872 PMC7603256

[39] Slotved HC, Kaltoft M, Skovsted IC, Kerrn MB, Espersen F. 2004. Simple, rapid latex agglutination test for serotyping of pneumococci (Pneumotest-Latex). J Clin Microbiol 42:2518–2522. doi:10.1128/JCM.42.6.2518-2522.2004. 15184429 PMC427861

[40] Vogels CBF, Watkins AE, Harden CA, Brackney DE, Shafer J, Wang J, Caraballo C, Kalinich CC, Ott IM, Fauver JR, et al. 2021. SalivaDirect: a simplified and flexible platform to enhance SARS-CoV-2 testing capacity. Med 2:263–280. doi:10.1016/j.medj.2020.12.010. 33521748 PMC7836249

[41] Allicock OM, Yolda-Carr D, Earnest R, Breban MI, Vega N, Ott IM, Kalinich C, Alpert T, Petrone ME, Wyllie AL. 2021. Method versatility in RNA extraction-free PCR detection of SARS-CoV-2 in saliva samples. bioRxiv. doi:10.1101/2021.12.27.21268334. PMC10290768 37369293

