# Supplementary material for: Persistence of Pneumococcal Carriage among Older Adults in the Community despite COVID-19 Mitigation Measures
Source: Microbiol Spectr. 2023 Apr 10;11(3):e04879-22. doi: 10.1128/spectrum.04879-22 (PMC10269788; doi:10.1128/spectrum.04879-22)
Supplement: Supplemental file 1 — Supplemental material. Download spectrum.04879-22-s0001.pdf, PDF file, 1.0 MB [file spectrum.04879-22-s0001.pdf]

APPENDIX

Persistence of pneumococcal carriage among older adults in the community despite COVID-19 mitigation measures

Anne L. Wyllie[a*#], Sidiya Mbodj[a], Darani A. Thammavongsa[a], Maikel S. Hislop[a], Devyn Yolda-Carr[a], Pari Waghela[a], Maura Nakahata[a], Anne E. Stahlfeld[a], Noel J. Vega[a], Anna York[a], Orchid M. Allicock[a], Geisa Wilkins[b], Andrea Ouyang[b], Laura Siqueiros[b], Yvette Strong[b], Kelly Anastasio[b], Ronika Alexander-Parrish[c], Adriano Arguedas[c], Bradford D. Gessner[c], Daniel M. Weinberger[a]

[a]Department of Epidemiology of Microbial Diseases, Yale School of Public Health, New Haven, CT 06510, USA

[b]Yale Center for Clinical Investigation, New Haven, CT 06510, USA

[c]Medical and Scientific Affairs, Pfizer Inc, 500 Arcola Rd, Collegeville, PA, 19426, USA.

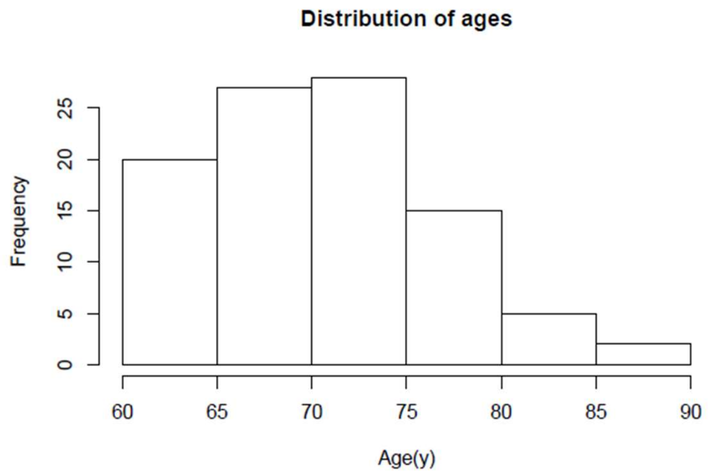

Supplementary Figure 1. Age distribution of study participants.

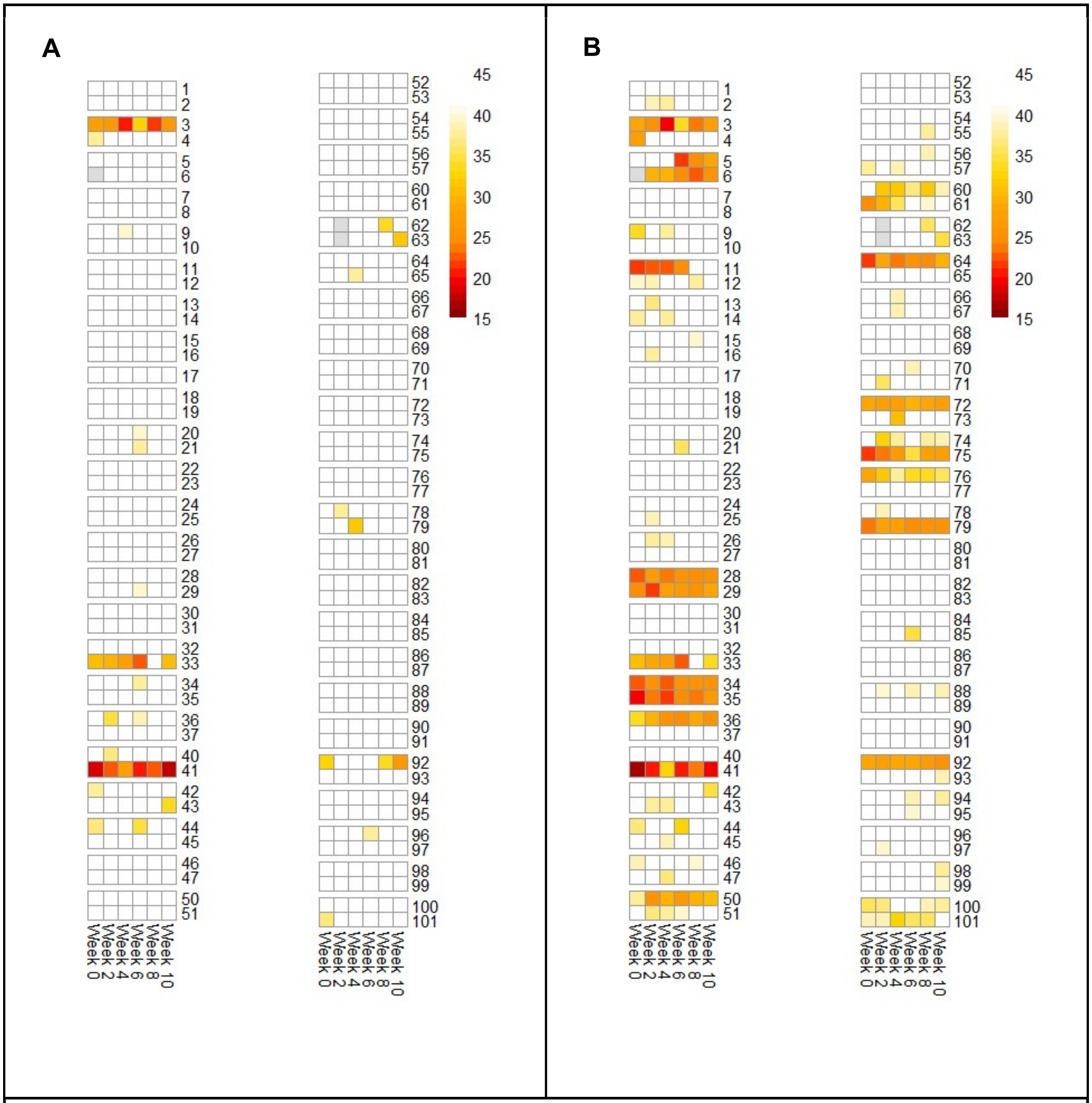

Supplementary Figure 2. Detection of pneumococcal genes, (A) *piaB* and (B) *lytA* in saliva samples from household pairs aged 60 years and older, collected December 2020 through June 2021. Each row represents a study participant, and each column is a sample (each collected 2 weeks apart). Darker colors indicate higher density of bacteria (lower PCR Ct value), white indicates no detection of the gene target, gray indicates the sample was not tested.

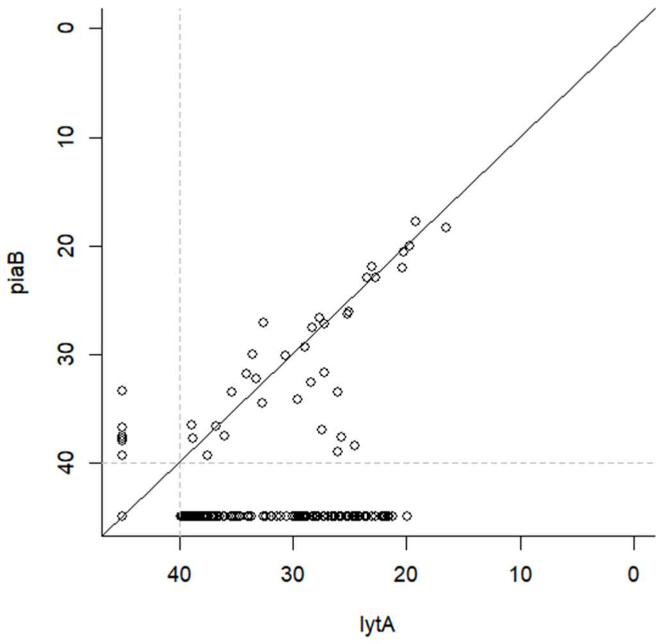

Supplementary Figure 3. PCR Ct values for pneumococcus genes *piaB* and *lytA* detected in saliva samples from household pairs aged 60 years and older, collected December 2020 through June 2021. Values along the diagonal line indicate equal concentrations for both gene targets.

FIRST VISIT QUESTIONNAIRE

Visit location

1. YCCI
2. Nursing home (Branford hills)
3. Assisted living facility
4. Private residence
5. Community center

Other: ______________________________

PARTICIPATION CRITERIA

Inclusion Criteria

1. Have you signed and dated the informed consent form and received a copy for your records? ☐ Yes ☐ No
2. Are you 60 years of age or older? ☐Yes ☐No

   If yes, what is your date of birth __________________________ (DD-MM-YYYY)
3. Are you experiencing any of the following symptoms **today**?
   a. Nasal congestion ☐Yes ☐No
   b. Coughing ☐Yes ☐No
   c. Running nose ☐Yes ☐No
   d. Sore throat ☐Yes ☐No
   e. Fever ☐Yes ☐No
   f. Other nose, throat or chest symptoms ☐Yes ☐No

      (List any other symptoms here_______________________________________)
4. Are you enrolling into this study together with a household member who is also 60 years of age or older? ☐Yes ☐No

Exclusion Criteria

1. Have you had a positive test for COVID?

   ☐Not tested ☐ No, it was negative ☐ Yes, it was positive

   If yes, when did you have this positive test? ____________________(DD-MMM-YYYY)
2. Have you received the pneumonia vaccine? ☐Yes ☐No

   If yes, when did you receive this vaccine? _____________________(DD-MMM-YYYY)

   Where did you receive this vaccine?

   1. Doctor's Office (name of Provider)_________________________________
   2. Hospital Stay
   3. Pharmacy
   4. Health Department
   5. Health Fair
   6. Senior Center
   7. Other __________________________
3. Have you been hospitalized for pneumonia in the last month (30 days)?

   ☐Yes ☐No If yes, when were you discharged? __________ (DD-MMM-YYYY)
4. Have you taken any antibiotics in the last month (30 days)?

☐Yes ☐No If yes, please describe_______________________

5. Does anyone under the age of 60 years live in your household ☐Yes ☐No

➔ **IF YES TO ANY IN THE LAST 4 WEEKS SAMPLE 4 WEEKS FROM THAT DATE**

DEMOGRAPHIC DATA

Weight: _______ **Height (feet, inches):** _____

Confirm participant gender: M / F / other________

What is your highest level of education?: ___________________________________

How would you describe your ethnicity?

1. American Indian or Alaska Native
2. Asian
3. Black or African American
4. Native Hawaiian or Other Pacific Islander
5. White
6. Unknown/Other: ____________________

Would you consider yourself Hispanic or Non-Hispanic? ☐ Hispanic ☐ Non-Hispanic

What is the relationship between yourself and the person you are enrolling into the study with?

1. Married couple
2. Unmarried couple
3. Roommate
4. Family member
5. Paid care provider
6. Other

How long have you been living together? Years_______ and months_______

How much time do you spend together each day?: __________hours

Are you <u>both</u> available to participate in the study every 2 weeks for the next three months?

☐Yes ☐No If no, when will you be able to do this? ______________

→ **SAMPLE AT A LATER DATE**

MEDICAL HISTORY

Have you received the flu shot this season?

☐Yes ☐No ☐Don't Know If yes, when? _______________ (DD-MM-YYYY)

Where did you receive this vaccine?

Yale University
HIC#: 2000026100

FORNIGHTLY VISIT (NUMBER) ______
MRN: ____________________________
Date of visit: _________ (DD/MMM/YYYY)

1. Doctor's Office (name of Provider)__________________________________
2. Hospital Stay
3. Pharmacy
4. Health Department
5. Health Fair
6. Senior Center
7. Other ________________________

Have you had any of the following symptoms in the past 2 weeks?

a. Nasal congestion ☐Yes ☐No
b. Coughing ☐Yes ☐No
c. Running nose ☐Yes ☐No
d. Sore throat ☐Yes ☐No
e. Fever ☐Yes ☐No
f. Other nose, throat or chest symptoms ☐Yes ☐No
(List any other symptoms here____________________________________)

Have you taken any medications for or been told by a health care provider that you have any of the following conditions?

1. Immunodeficiency (Examples include: HIV/AIDS, corticosteroid medication like "Prednisone", medicine or therapy to treat a cancerous tumor or mass, have had your spleen removed or have chronic kidney disease) ☐Yes ☐No ☐Don't Know
2. Diabetes ☐Yes ☐No ☐Don't Know
3. Chronic heart disease:
   a. Congestive heart failure (CHF) ☐Yes ☐No ☐Don't know
   b. Enlarged or thick heart muscle ☐Yes ☐No ☐Don't know
4. Chronic liver disease:
   a. Liver cirrhosis ☐Yes ☐No ☐Don't know
   b. Liver cancer ☐Yes ☐No ☐Don't know
5. Chronic lung disease:
   a. chronic obstructive pulmonary disease (COPD) ☐Yes ☐No ☐Don't Know
   b. Emphysema ☐Yes ☐No ☐Don't know
   c. Asthma ☐Yes ☐No ☐Don't know

Do you currently smoke cigarettes or e-cigarettes?

☐ No ☐ Yes, cigarettes ☐ Yes, e-cigarettes

How many cigarettes do you smoke a day? Enter number: _________________

Yale University
HIC#: 2000026100

FORNIGHTLY VISIT (NUMBER) ______
MRN: ____________________________
Date of visit: _________ (DD/MMM/YYYY)

Have you ever smoked cigarettes in the past? ☐ No ☐ Yes, when did you quit?

☐ In the last year ☐ In the last 5 years ☐ 10 or more years ago

Do you have any regular contact with children? ☐ No ☐ Yes

What is the age range of the children you have had contact with?

☐ <12 months ☐ 13-23 months ☐ 24-59 months

☐ 5-10 years ☐ >10 years Record actual age if given: _____________

How often do you usually have contact with children?

☐ Daily ☐ Every few days ☐ Once or twice a month

How much contact per day do you have?

1. <4 hours (morning or afternoon or evening)
2. 4-8 hours (full day)
3. 8+ hours (longer day care/overnight)

Time of sample collection: _____________________________

Yale University
HIC#: 2000026100

FORNIGHTLY VISIT (NUMBER) ______
MRN: ____________________________
Date of visit: _________ (DD/MMM/YYYY)

FORTNIGHTLY VISITS QUESTIONNAIRE

Visit location

4. YCCI
5. Nursing home (Branford hills)
6. Assisted living facility

4. Private residence
5. Community center

Other: ___________________________

Have you taken part in any social activities or outings during the past two weeks?

☐ No ☐ Yes, **what sorts of activities have you participated in?:**

☐ Activities at community centers ☐ Activities with family

☐ Activities with friends ☐ Fitness activities

Other: ____

Have you had any contact with children in the past two weeks? ☐ No ☐ Yes

If yes, what is the age range of the children you have had contact with?

☐ <12 months ☐ 13-23 months ☐ 24-59 months

☐ 5-10 years ☐ >10 years Record actual age if given: _____________

How often do you usually have contact with children?

☐ Daily ☐ Every few days ☐ Once or twice a month

How much contact per day do you have?

4. <4 hours (morning or afternoon or evening)
5. 4-8 hours (full day)
6. 8+ hours (longer day care/overnight)

Have you had been tested for COVID in the last two weeks?

☐ No ☐ Yes, it was negative ☐ Yes, it was positive

Have you had any of the following symptoms in the past 2 weeks?

g. Nasal congestion ☐ Yes ☐ No
h. Coughing ☐ Yes ☐ No
i. Running nose ☐ Yes ☐ No
j. Sore throat ☐ Yes ☐ No
k. Fever ☐ Yes ☐ No

FORNIGHTLY VISIT (NUMBER) ______
MRN: ____________________________
Date of visit: _________ (DD/MMM/YYYY)

I. Other nose, throat or chest symptoms ☐Yes ☐No
(List any other symptoms here____________________________________)

Which symptoms are you experiencing <u>today</u>?

a. Nasal congestion ☐Yes ☐No
b. Coughing ☐Yes ☐No
c. Running nose ☐Yes ☐No
d. Sore throat ☐Yes ☐No
e. Fever ☐Yes ☐No
f. Other nose, throat or chest symptoms ☐Yes ☐No
(List any other symptoms here____________________________________)

Have you had any sick visits to the doctor or been hospitalized in the last two weeks?

☐No ☐ Yes ☐ Yes, more than once

Have you taken any antibiotics in the last two weeks?

☐No ☐Yes If yes, please describe_______________________

Have you received any new vaccines in the last two weeks?

☐ Flu vaccine ☐ Pneumonia vaccine ☐ Other vaccine, describe: ____________

Where did you receive this vaccine?

1. Doctor's Office (name of Provider)_________________________________
2. Hospital Stay
3. Pharmacy
4. Health Department
5. Health Fair
6. Senior Center
7. Other ________________________

Time of sample collection: _______________________

Reason for sample refusal or study withdrawal: ________________________________

<u>ON FINAL VISIT:</u> Do you give us your permission to re-contact you to participate in this study again next year, or in other studies that may interest you? ☐ No ☐ Yes
